# Supplementary material for: Label‐free discrimination of extracellular vesicles from large lipoproteins
Source: J Extracell Vesicles. 2023 Jul 25;12(8):12348. doi: 10.1002/jev2.12348 (PMC10366660; doi:10.1002/jev2.12348)
Supplement: Supplementary file 1 [file JEV2-12-12348-s001.pdf]

Interferometric nanoparticle tracking analysis enables label-free discrimination of extracellular vesicles from large lipoproteins.

Anna D. Kashkanova,[1,2†] Martin Blessing,[1,2,3†], Marie Reischke[1], Jan-Ole Baur[4*],
Andreas S. Baur[4], Vahid Sandoghdar[1,2,3*] and Jan Van Deun[4*]
[1]Max Planck Institute for the Science of Light, 91058 Erlangen, Germany
[2]Max-Planck-Zentrum für Physik und Medizin, 91058 Erlangen, Germany
[3]Department of Physics, Friedrich-Alexander-Universität Erlangen-Nürnberg, 91058 Erlangen, Germany
[4] Department of Dermatology, Universitätsklinikum Erlangen, Friedrich-Alexander-Universität Erlangen-Nürnberg, 91052 Erlangen, Germany
[†] Equal contributions
[*] Corresponding authors. Emails: Vahid.Sandoghdar@mpl.mpg.de; Jan.VanDeun@uk-erlangen.de

Supplemental figures

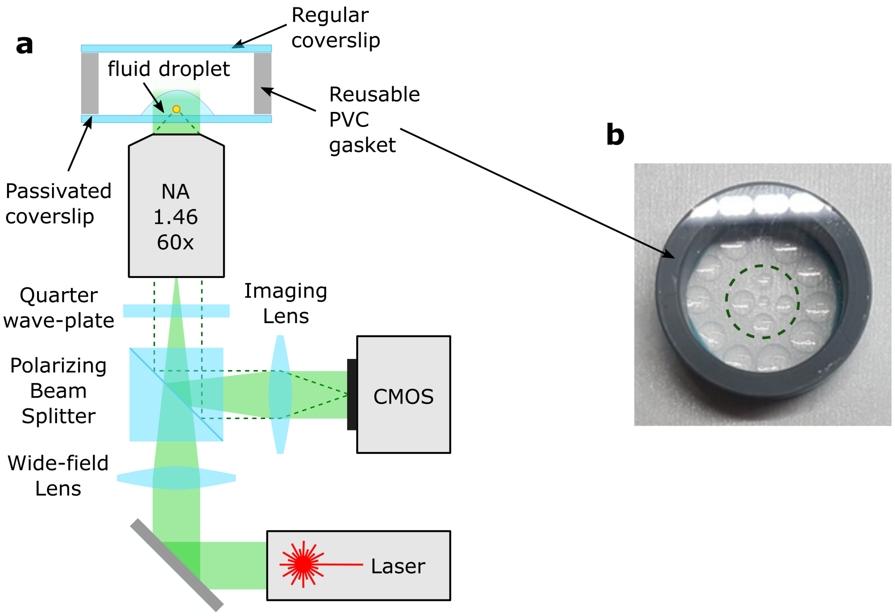

Figure S1: iNTA measurement setup. **(a)** Wide-field iSCAT setup for tracking freely diffusing particles. Linearly polarized light from a laser passes a polarizing beam splitter followed by a $\lambda/4$ plate that renders its polarisation circular. A wide-field lens ($f = 400$ mm) focuses the light at the back focal plane of the objective. An imaging lens ($f = 500$ mm) projects the reflected (solid green area) and scattered (dashed line) light on a CMOS camera chip.**(b)** A PVC chamber used for the measurements. A reusable PVC gasket is glued using dental glue to a coverslip passivated with Sil-PEG (see Methods). The droplets containing the sample are positioned in the center of the coverglass (inside dashed green circle), here shown with volumes ranging from 200 nl up to 2 μl. For our measurements, we worked with volumes from 500 nl up to 1 μl. On the perimeter several large droplets of PBS are positioned to serve as a "reservoir" to keep humidity inside the chamber high and prevent evaporation. Another coverslip is positioned on top of the PVC gasket.

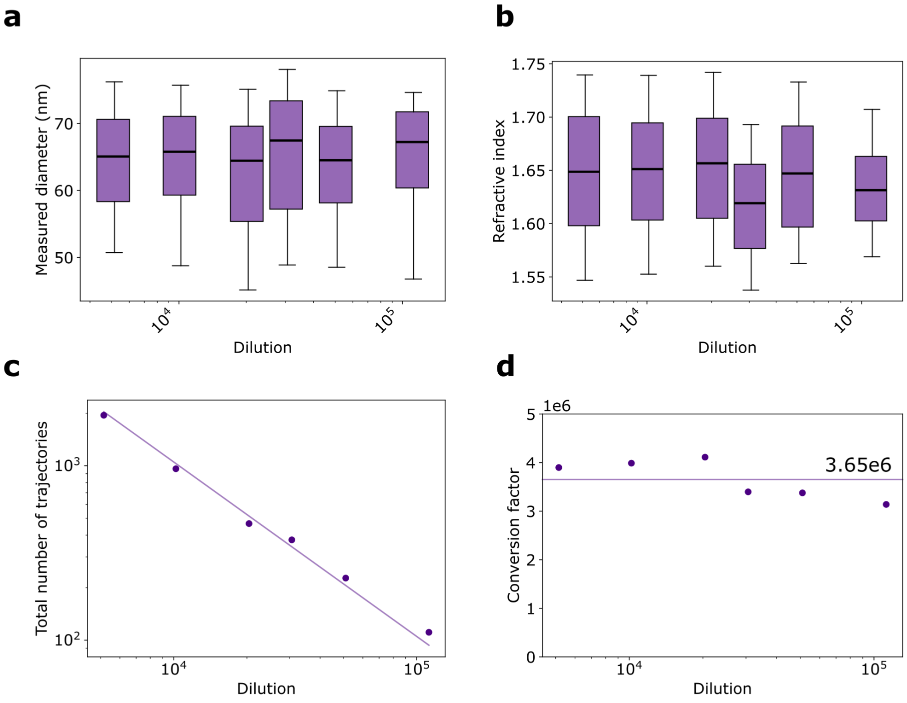

Figure S2: iNTA particle concentration calibration. A sample of 60 nm polystyrene spheres was measured with iNTA at different dilutions factors (DF) from 5000x to 100,000x. The measured **(a)** size and **(b)** refractive index of particles at different dilutions demonstrate the robustness of the method (median and 10-90 percentile (whiskers)). **(c)** the number of trajectories (N_{traj}) recorded over 10 minutes for different dilutions. Only particles that cross the focal plane (es evidenced by the width of the point spread function) are included. **(d)** Using the NTA measurement of particle concentration (C_{NTA}) for this sample we calculate the conversion factor as $C_{\mathrm{NTA}}/(N_{\mathrm{traj}} \times DF) = 3.65\mathrm{E}6$, which allows us to convert the number of trajectories into particle concentration.

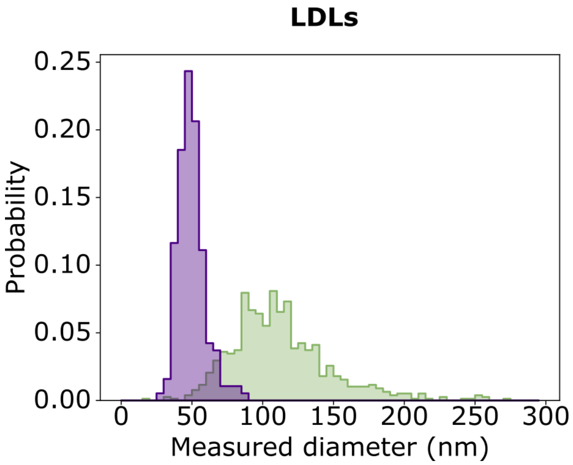

Figure S3: Low density lipoprotein size distribution. Measurement performed using NTA (green) and iNTA (purple). Both techniques measured particles of larger size than expected for LDLs (median size of 108 nm for NTA and 48 nm for iNTA), possibly representing large LPs contaminating the LDL preparation.

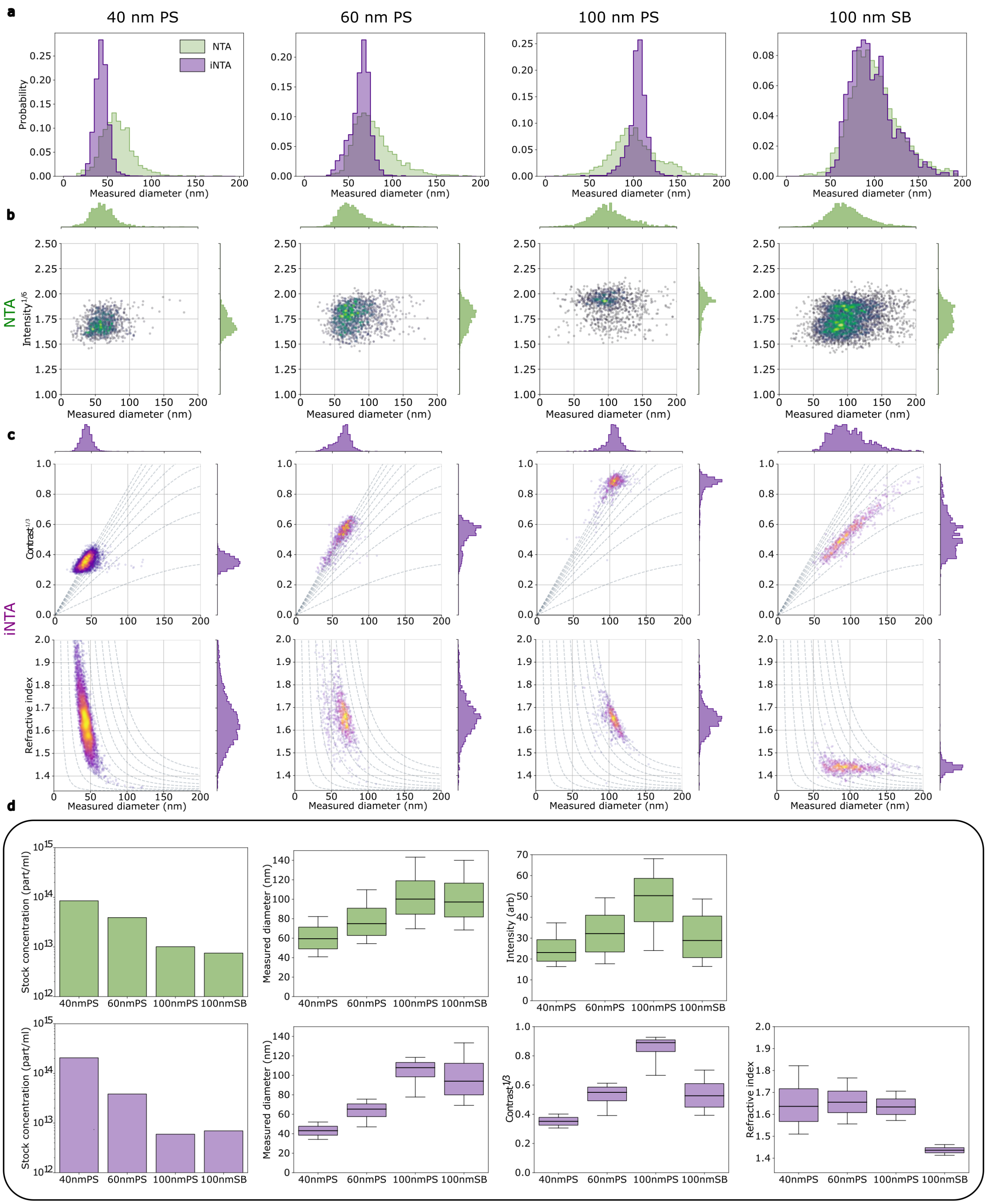

Figure S4: Performance comparison of NTA and iNTA using synthetic beads. (a) 40, 60, 100 nm polystyrene spheres (PS) and 100 nm silica beads (SB) size distributions as measured by NTA (green) and iNTA (purple). For iNTA measurements, stock concentrations were diluted by a factor of 1E4. For NTA, dilution factors were 1E6 for 40 and 60 nm beads, and 1E5 for 100 nm PS and SB beads. **(b)** NTA plots of intensity$^{1/6}$ vs. size. The intensity is plotted to the power of 1/6 because in dark field measurement it is expected to scale as a sixth power of diameter. Blue tones indicate lower density of points and yellow tones indicate higher density of points. **(c)** iNTA plots of contrast vs. size (top row) and refractive index vs. size (bottom row). Purple tones indicate lower density of points and yellow tones indicate higher density of points. In the top row dashed gray lines indicate the constant refractive index ranging from 1.34 to 1.66 in steps of 0.04. In the bottom row the dashed line indicate lines of constant contrast$^{1/3}$ from 0.1 to 0.9 in steps of 0.1. Note that the extracted diameter and contrast are correlated, despite being measured independently. This confirms that both are extracted correctly. The effective refractive index for small polystyrene spheres has a broader size distribution than for large polystyrene spheres and for silica beads. This is caused by the closer spacing of refractive index lines on size/contrast plot for smaller particles and larger RI. **(d)** Summary plots for NTA (top) and iNTA (bottom). Concentration is displayed as mean, diameter/contrast/intensity as median and 10-90 percentile (whiskers).

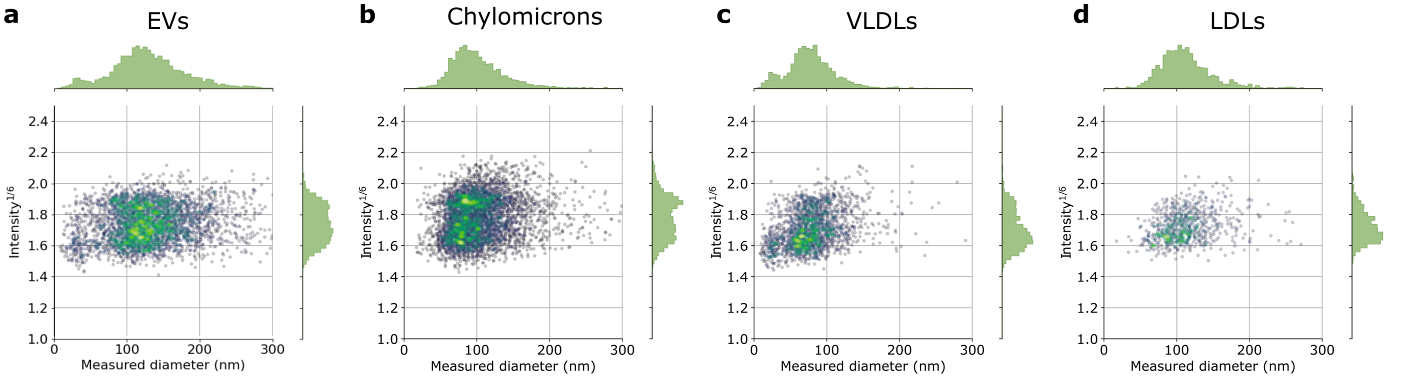

Figure S5: NTA measurements of EVs and lipoproteins. The scattered intensity returned by the instrument is plotted vs. the measured diameter. The blue tones correspond to lower point density while the yellow tones indicate higher point density. The samples are **(a)** EVs, **(b)** ULDLs, **(c)** VLDLs, and **(d)** LDLs.

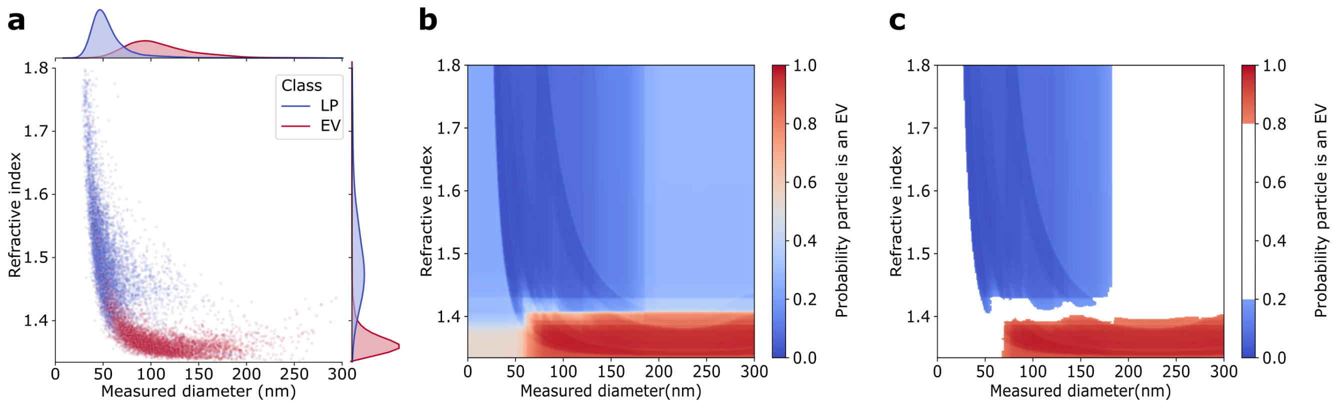

Figure S6: Random forest particle classifier to differentiate EVs and lipoproteins. **(a)** Training dataset with two separate measurements of EVs and lipoproteins. **(b)** Resulting probability map returned by the classifier trained using python scikit-learn package. The features used by the classifier are particle size, maximum iSCAT contrast to the power of 1/3 and refractive index. The parameters are as follows: 500 estimators, balanced class weight, 10 samples required to be at a leaf node minimum and maximum depth of the tree of 5. The other parameters are kept at their default values. **(c)** Same map as in (b) but only regions with $\geq 80\%$ confidence of particle belonging to either class are plotted.

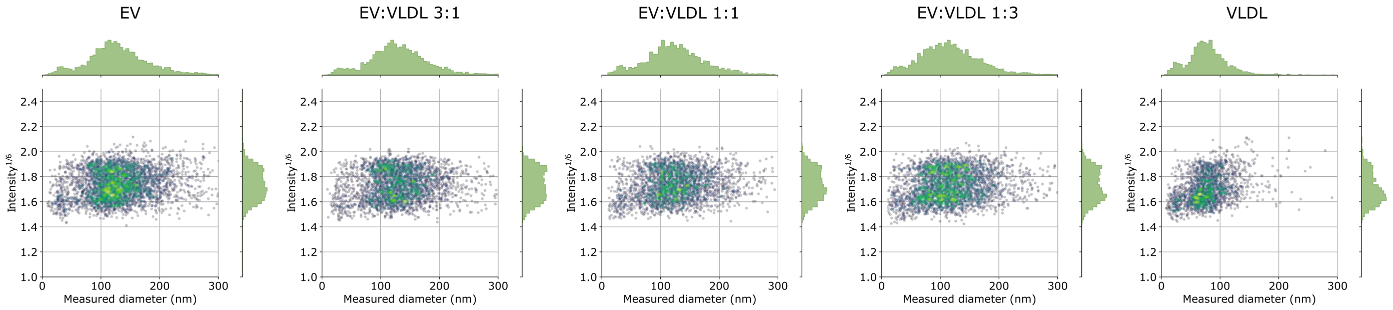

Figure S7: NTA measurements of EV-VLDL mixtures. The blue tones correspond to lower point density while the yellow tones indicate higher point density.

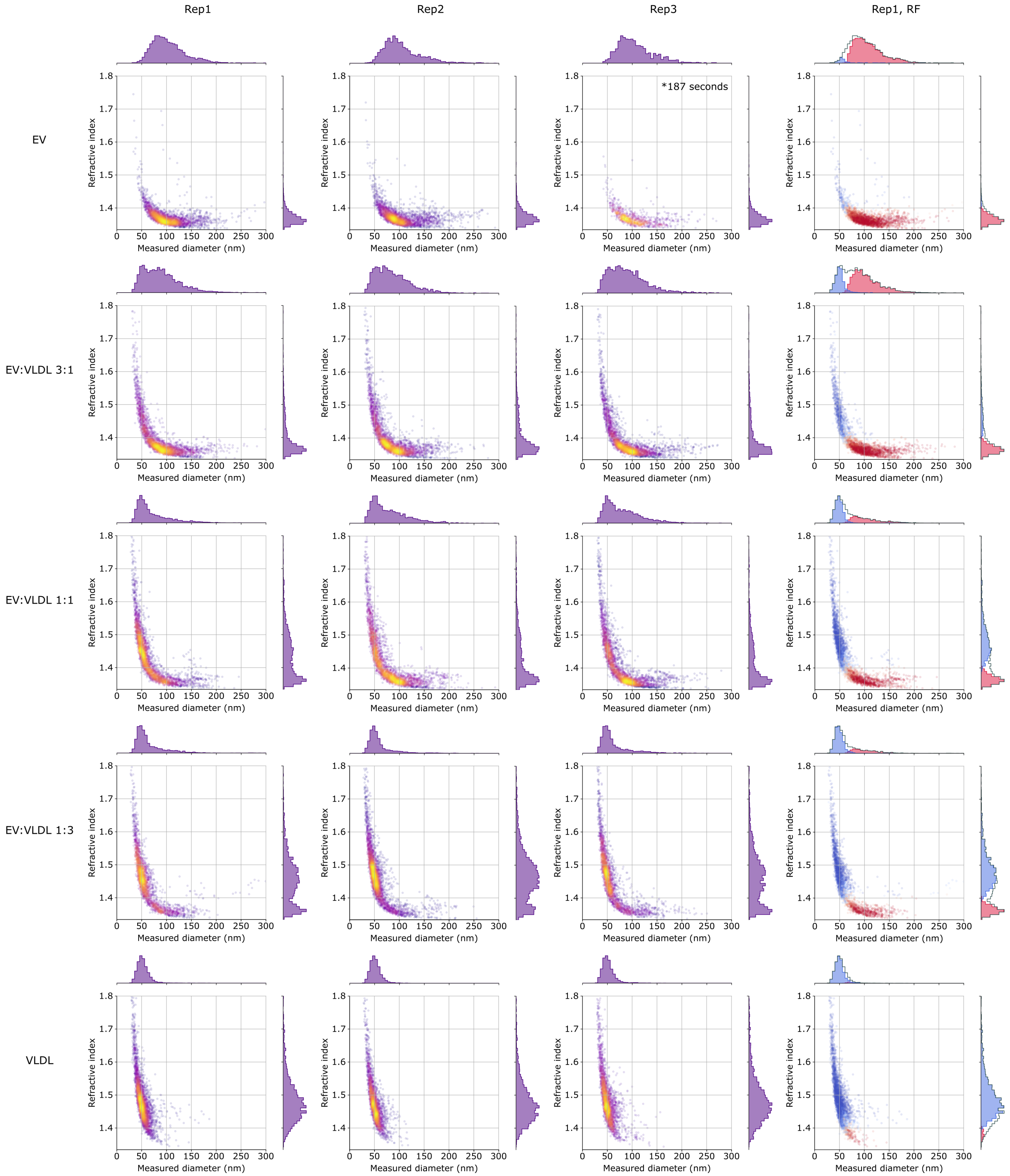

Figure S8: iNTA measurements of EV-VLDL mixtures. The three left columns show technical replicates of iNTA measurements of EV-VLDL mixes. All samples except for Rep3-EV were measured for 10 minutes. The right column demonstrates the application of random forest classifier to Rep 1.

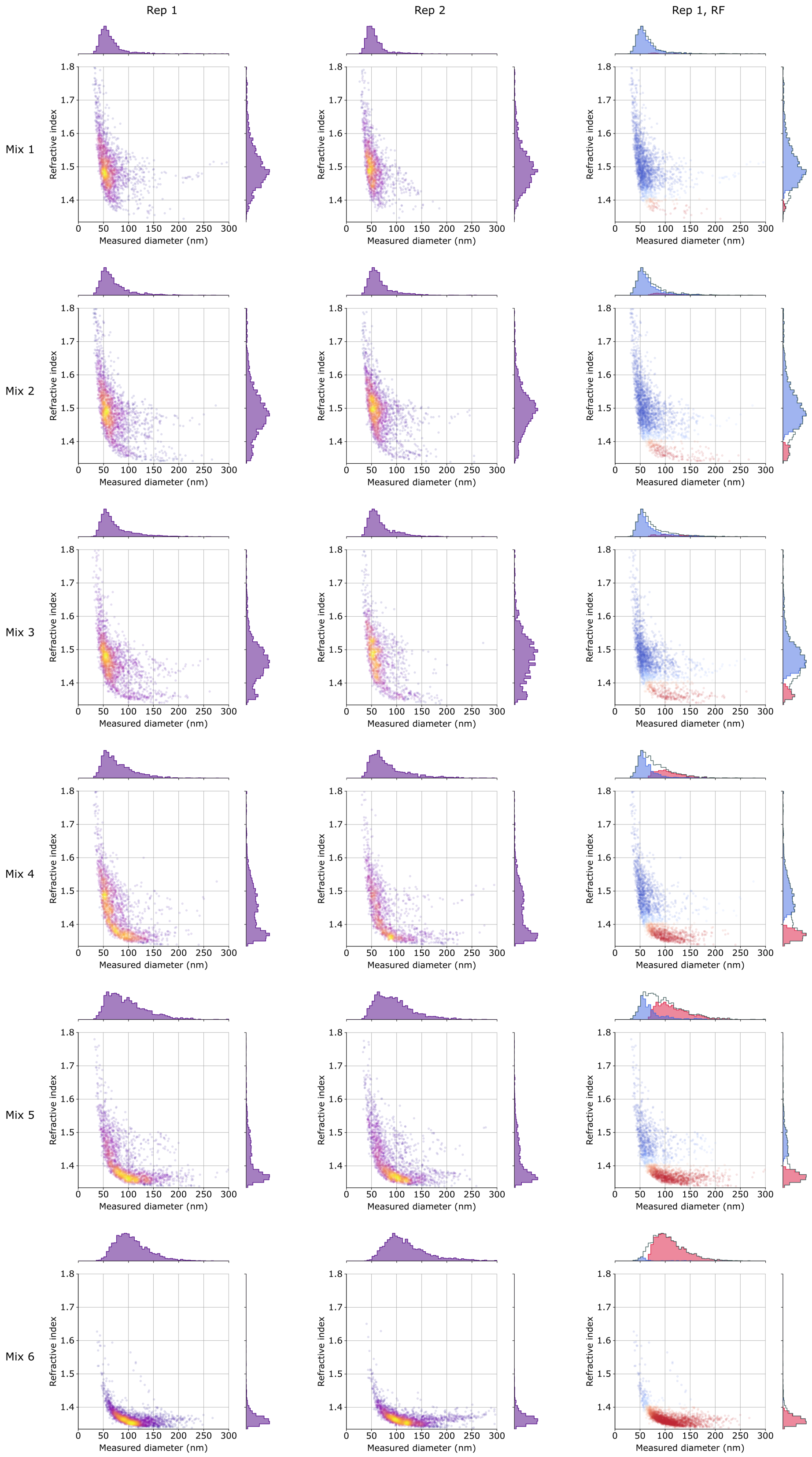

Figure S9: iNTA measurements of EV-lipoprotein mixtures. The two left columns show technical replicates of iNTA measurements of EV-LP mixes. All samples were measured for 10 minutes. The right column demonstrates the application of random forest classifier to Rep 1.

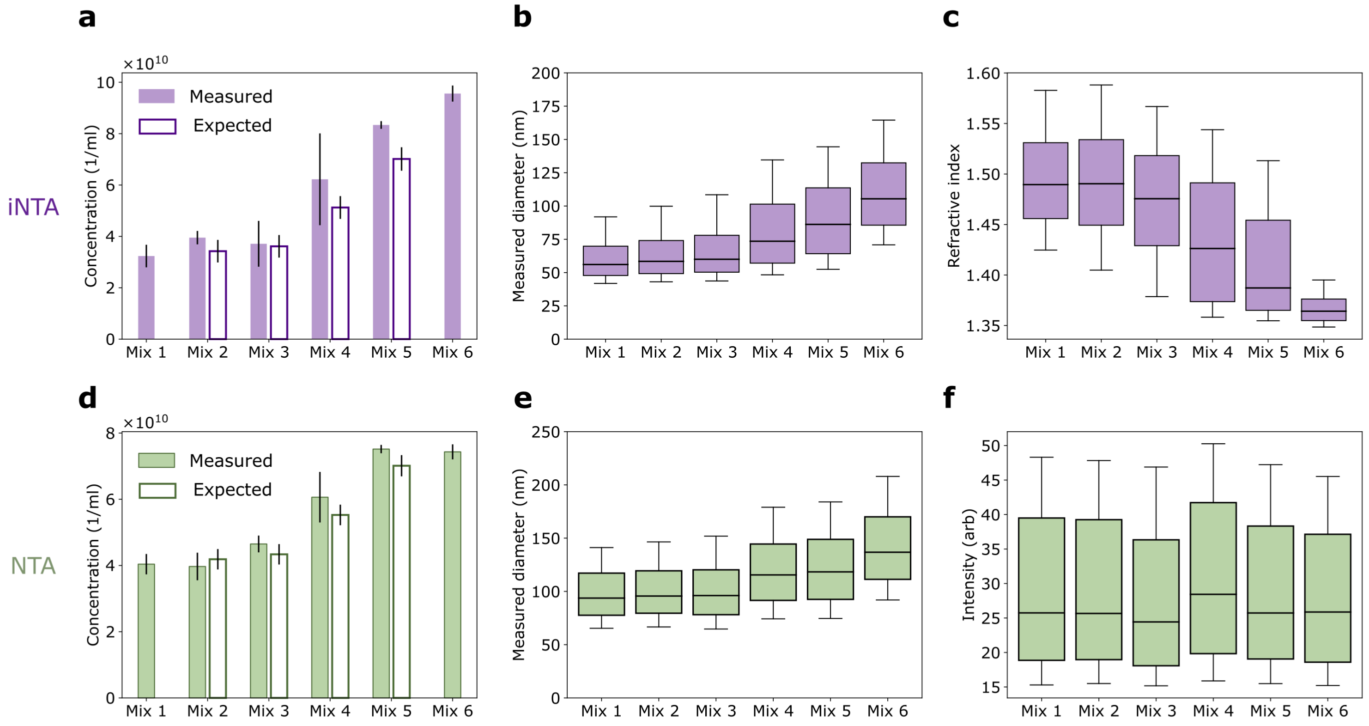

Figure S10: Summary of iNTA and NTA measurements of EV-lipoprotein mixtures. **(a)** Total particle concentration (mean and standard deviation), **(b)** particle diameter (median and 10-90 percentile) and **(c)** effective refractive index (median and 10-90 percentile) as measured by iNTA. **(d)** Total particle concentration (mean and standard deviation), **(e)** particle diameter (median and 10-90 percentile) and **(f)** scattering intensity (median and 10-90 percentile) as measured by NTA. The expected values in (a) and (d) are calculated using the measured values of Mix 1 (pure lipoproteins) and Mix 6 (pure EVs).

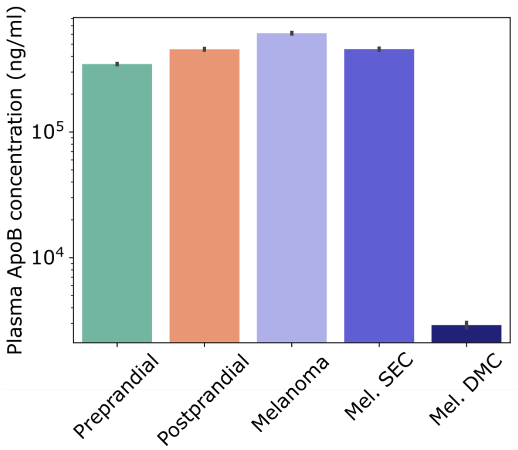

Figure S11: ApoB content as measured by ELISA in pre- or postprandial plasma of a healthy volunteer, and lipemic melanoma patient plasma. Mean and standard deviation of duplicate measurements are shown. Measurement of ApoB in DG processed sample was below the limit of detection.

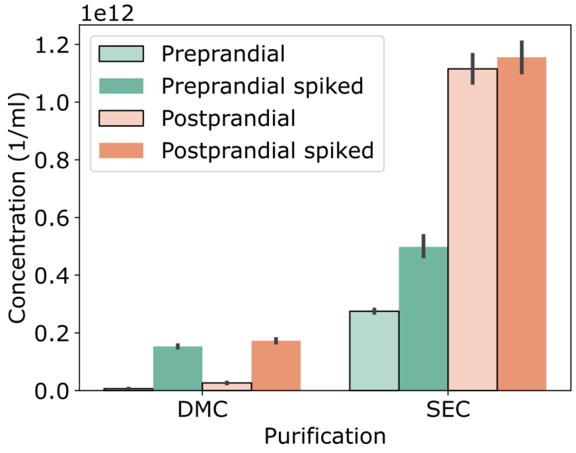

Figure S12: NTA concentration measurements of pre- and post-prandial plasma processed by DMC or SEC, without or with addition of an exogenous EV spike. Mean and standard deviation are shown.

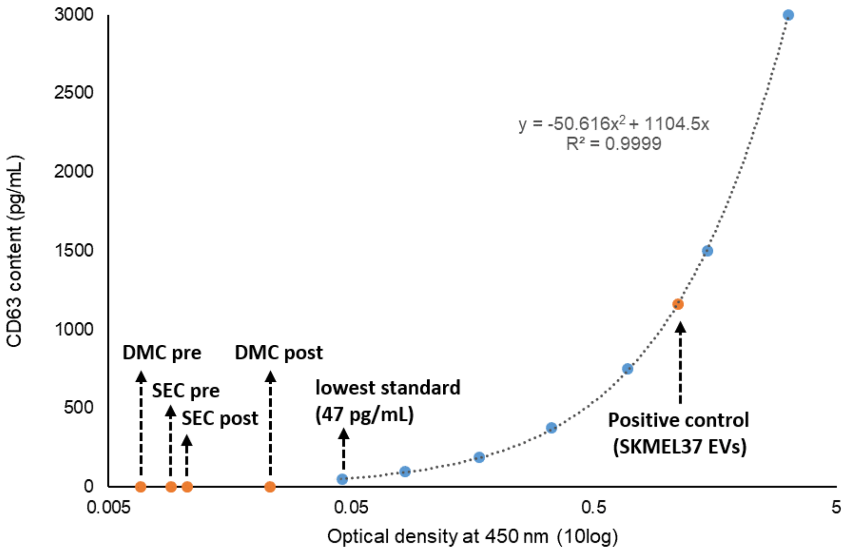

Figure S13: CD63 ELISA. Pre- and post-prandial plasma of a healthy volunteer was processed by SEC and DMC, and CD63 content measured as a proxy of EV presence. All samples were below limit of detection (i.e. an OD450 below the lowest standard). SKMEL-37 EVs were used as positive control.

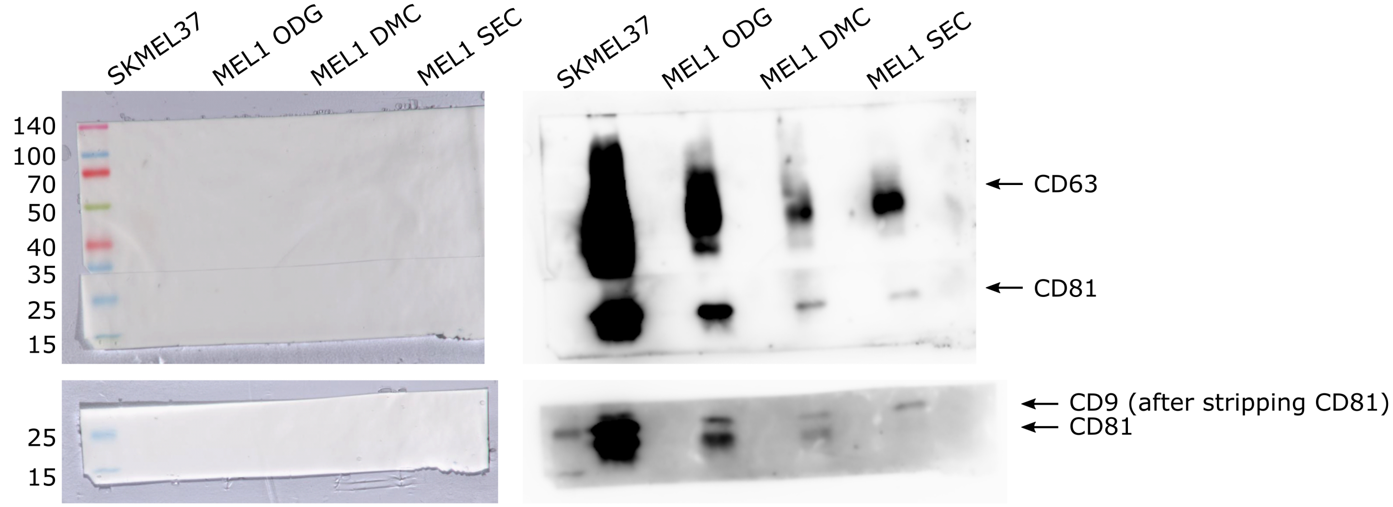

Figure S14: Full-length Western blot corresponding to Figure 4a. Normal light pictures of the membranes (left) are shown side-by-side with chemiluminescence images (right). The membrane was cut between CD63 and CD81, to allow staining with both antibodies simultaneously. The lower membrane piece was stained for CD9 after stripping, as described in the Methods section.

1 Supplemental information

1.1 Synthetic nanoparticle specifications

The following types of nanoparticles were used for instrument characterization:

- 100nm polystyrene: Thermo Fisher, Cat. No.: 3100(-008), Lot: 229003, Certified Mean Diameter: 100nm ± 4nm, k=2 [TEM], Coefficient of Variation: 7.7%, Hydrodynamic radius: 98-104nm [PCS], RI: 1.59 @589nm; Density: 1.05 g/cm3; Approximate concentration: 1% solids
- 60nm polystyrene: Thermo Fisher, Cat. No.: 3060(-008), Lot: 228547, Certified Mean Diameter: 61nm ± 4nm, k=2, Coefficient of Variation: 15.6%, Hydrodynamic radius: 58-68nm [PCS], RI: 1.59 @589nm; Density: 1.05 g/cm3; Approximate concentration: 1% solids
- 40nm polystyrene: Thermo Fisher, Cat. No.: 3040(-004), Lot: 230327, Certified Mean Diameter: 41nm ± 4nm, k=2 [PCS], Coefficient of Variation: not determined, RI: 1.59 @589nm; Density: 1.05 g/cm3; Approximate concentration: 1% solids
- 100nm silica: Corpuscular, Cat. No.: 140120-10, Lot: NX731, Mean Diameter: 101.9nm, Polydispersity index: 0.02

TEM=Transmission electron microscopy
PCS=Photon correlation spectroscopy

NB: While the manufacturer of polystyrene beads specifies how the size is measured, they do not specify how the RI, density and concentration are measured nor the precision of those values.

1.2 Characterizing precision/spread of the refractive index values

We have characterized the precision/spread of the refractive index (RI) values, by performing a simulation as follows:

1. We recorded 100 videos at the same illumination intensity as was used for the measurements, but with pure water as the sample.
2. We added iSCAT point spread functions[1] to the videos, simulating diffusing nanoparticles with a given scattering cross-section and ensuring that they cross the focal plane.
3. We analyzed the videos and extracted the maximum contrast for each diffusing particle. The relative standard deviation for each mean maximum contrast (over 100 particles) is shown in Fig. S15a. As expected, due to photon shot noise, the relative standard deviation is larger for particles with low iSCAT contrast (which, since we have the same illumination intensity, translates to lower SNR).
4. We used the maximum contrast values to calculate RI values, given the particle size as described in our previous work[2]
5. For each combination of size and contrast, we calculate the median RI as well as the interquartile range (IQR) of RI.

Figure S15b shows the expected IQR given the particle's size and median RI. From this 2D plot, we can see that the RI spread is generally larger for small particles and smaller for large particles. It is also particularly small for large particles with small RI. We can now use this information to simulate the 2D diameter-refractive index plot for particles with an RI of 1.518 and similar size distribution to VLDLs, which is shown in Fig. S15c. We note that the RI reaches values of 1.8, as we also observed in the real distribution (Fig. 1b). Repeating this analysis for EVs results in a markedly narrower RI distribution, shown in Fig. S15d. In all these plots, the spread in the RI is exclusively due to photon shot noise, which affects contrast determination. Additional spread may be caused by errors in contrast determination due to other particles nearby, or due to errors in size determination. Note that in the real measurement the distribution is cut off by the contrast sensitivity threshold, for our setup: $\text{contrast}^{1/3} < 0.3$. For EVs and other kinds of vesicles, we expect the RI to be higher for small particles since the ratio of lipids to inner content is larger in that case (see Ref. 2, SI). It is possible that smaller lipoproteins have larger RI as well due to larger ratio of ApoB to lipid content.

In order to account for possible additional errors, we measured a sample with RI similar to that of VLDLs: a water suspension of immersion oil with nominal RI of 1.52. The sample was prepared by vortexing a drop of immersion oil in milliQ for 1 minute and then allowing it to settle for 10 minutes. The supernatant consisting of water with oil droplets was measured with iNTA. The results are shown in Fig. S15e,f. The size distribution of oil droplets and VLDLs is quite similar. The median RI of oil droplets is 1.53, while the IQR is 1.50-1.56 (0.06), which is slightly lower than for VLDLs. Here we also observe particles reaching the RI of 1.8. While we cannot make definite statements about "true" lipoprotein RI distribution, we demonstrate that for uniform oil droplets a comparable spread in RI is present.

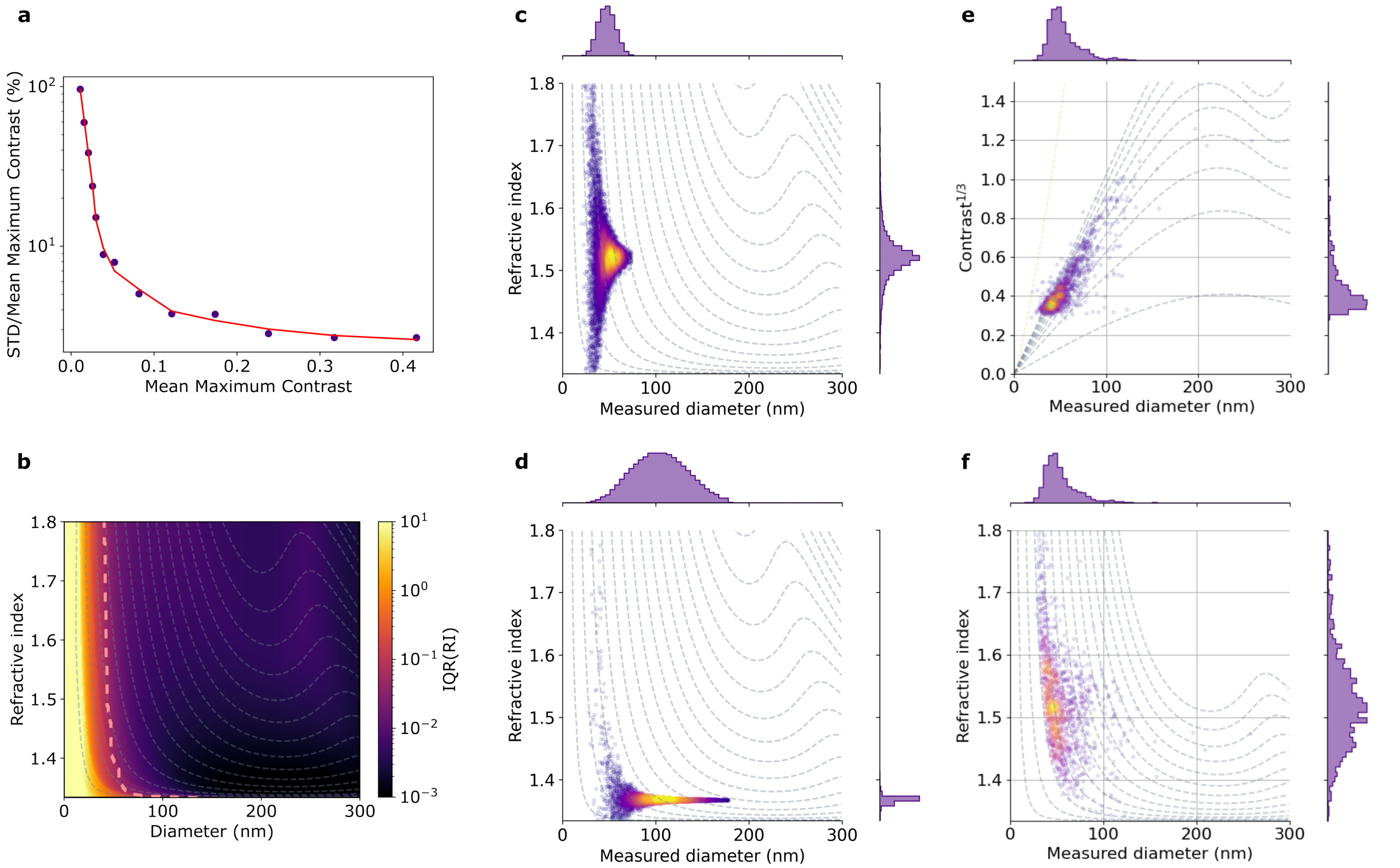

Figure S15: **(a)** The relative standard deviation for each mean maximum contrast (over 100 particles). The violet dots indicate the results of individual simulations, while the red line shows the result of applying the Savitzky–Golay filter. **(b)** The IQR of RI plotted vs the particle diameter and refractive index. The limits on the color map are set to 0.001 and 10, even though IQR can reach lower and higher values. To the left of the dashed red line the IQR is larger than 0.05. **(c)** The simulated RI distribution, assuming size distribution with the same mean and standard deviation as VLDLs. The refractive index is set to be 1.52. **(d)** Same as (c) but the size distribution is assumed to have the same mean and standard deviation as EVs. The refractive index is set to 1.37. **(e, f)** Contrast$^{1/3}$ and RI vs.diameter plots for oil nanodroplet suspension in milliQ. In (b), (c), (d), (f) dashed gray lines indicate the lines of constant contrast$^{1/3}$ starting from 0.1 and progressing in steps of 0.1. Note that the spacing of the lines in (f) is different from the rest, because for technical reasons the measurement was conducted on a different setup with a different scattering cross-section to contrast calibration factor. In (e) dashed gray lines indicate the lines of constant RI ranging from 1.34 to 1.66 in steps of 0.04.

1.3 Effect of focal plane position on particle size and contrast

Using 30 nm GNP, we characterized the dependence of particle size and contrast on the focal plane position. We used the focus lock and performed sets of 200 measurements at 17 different positions of the focal plane. The results are summarized in Fig. S16. The extracted particle size is very stable with the mean of 34.43 nm and standard deviation of 0.15 nm. As expected, the contrast stably decreases as the focal plane is moved further away from the coverglass because of a reduced overlap between the spherical waves scattered by the particle and the light reflected by the substrate. An outlier for the focal plane position at 0 (at the coverglass) is explained by the fact that no particles can possibly cross the focal plane and thus achieve maximum contrast. Due to the strong dependence of particle contrast (*i.e.* c scattering cross-section to contrast calibration factor) on the focal plane position, all measurements described in this manuscript were performed with a focal place fixed at 1 μm above the coverglass.

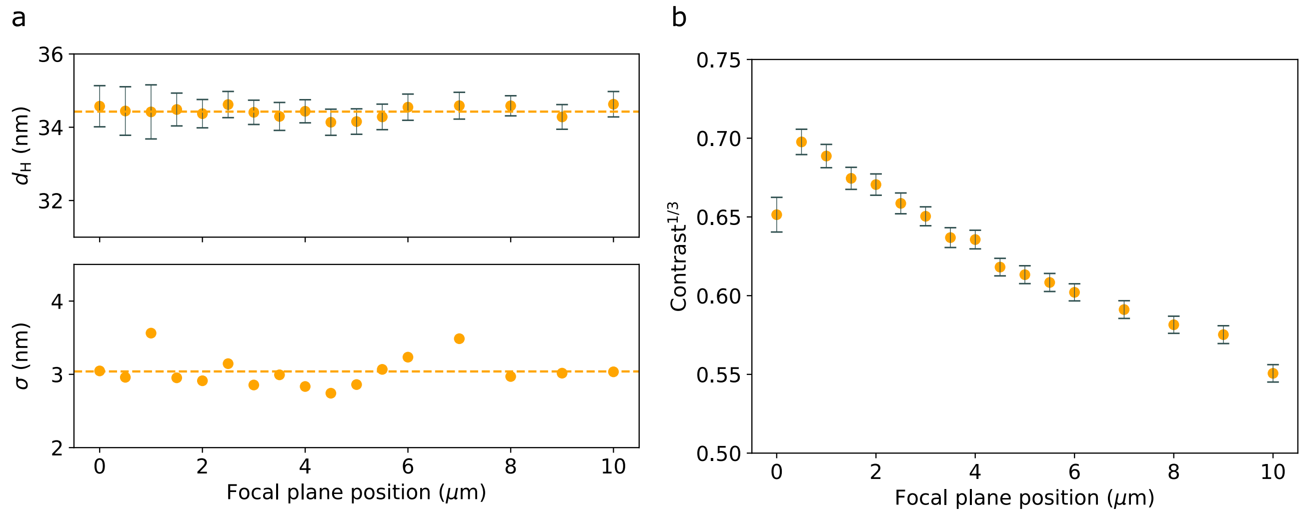

Figure S16: The extracted size and contrast of the 30 nm GNP for different positions of the focal plane relative to the coverglass.

1.4 Analysis of isolation method-dependent measurement artefacts

In Figures 3f and 4c of the main text, it looks like there is a small population of EVs in the DMC samples with a slightly higher RI and size. In order to verify that this population is not a result of DMC processing, we obtained density gradient-purified SKMEL37 EVs and measured them by iNTA before and after DMC processing. The particle distribution before DMC processing is shown in Fig. S17a. Figure S17b shows the particle distribution after DMC processing. We see that the two populations have nearly identical size and RI distributions, without any additional population. We have also repeated the DMC vs DG comparison for a different melanoma patient, to check whether we find a similar difference in EV populations. Interestingly, in the DG sample, there is now also a small 'extra' population of EVs visible (indicated by black arrows). This DG EV sample also appears to contain some more contaminants than the sample in manuscript Figure 4d, as suggested by a slightly higher population of lipoproteins. This, together with the finding that this population appeared after spiking of DMC samples with EVs in Figure 3f, leads us to hypothesize that these actually represent EVs attached to small LPs, which could theoretically increase their size and RI. This has been shown to happen for HDL and LDL.[3,4] That we did not detect this population in the artificial EV-LP mixtures could be due to incubation parameters (*e.g.* in Ref.3, EVs and LPs were incubated for 2h at 37°C, which we did not perform) or the necessity of other plasma proteins to facilitate this interaction (*e.g.* in the EV corona).

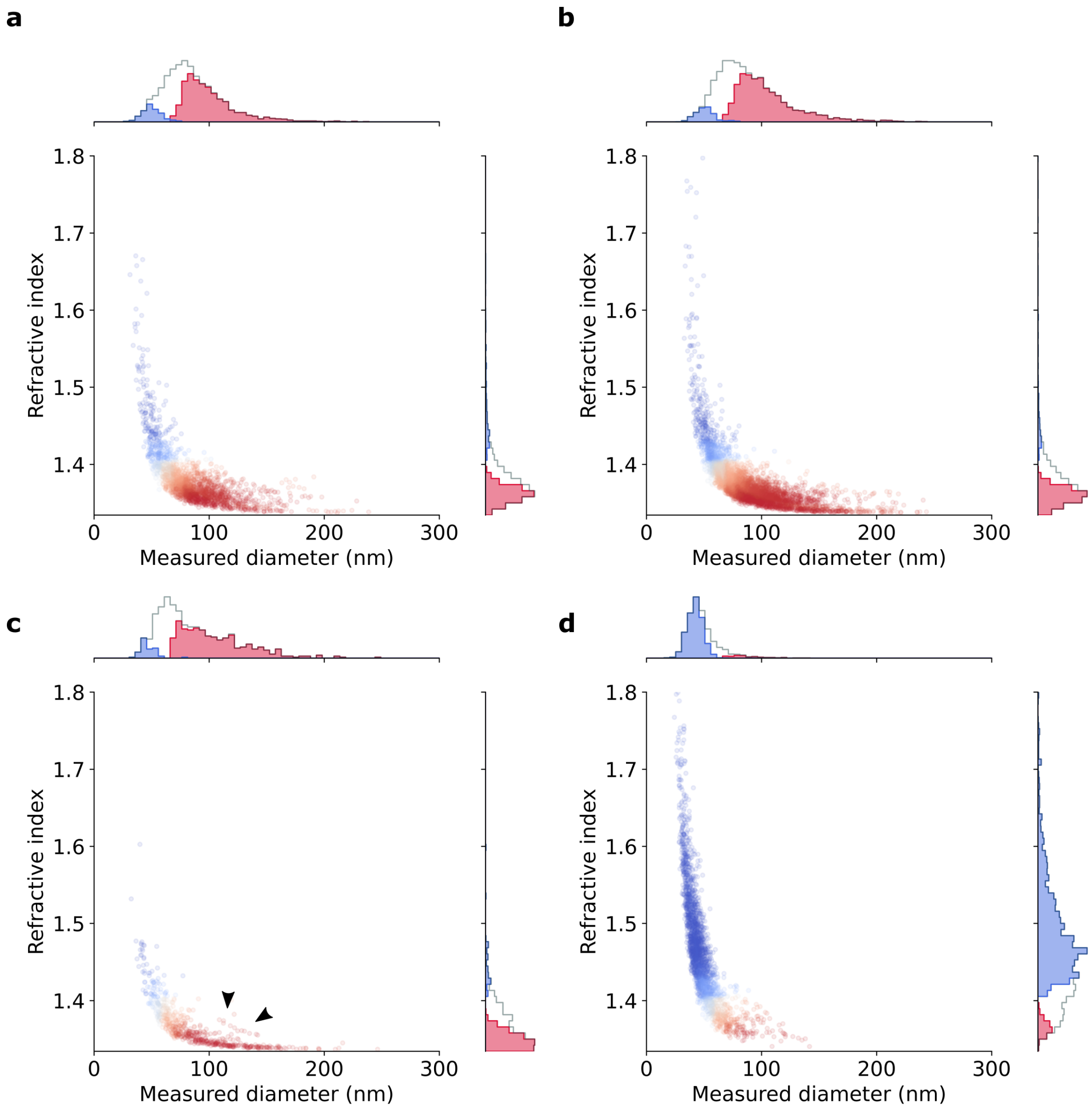

Figure S17: **(a)** Density gradient-purified SK-MEL37 EVs before DMC isolation versus **(b)** after DMC isolation. **(c)** EVs enriched from a melanoma patient plasma sample using DG. **(d)** Same plasma sample as (c), processed by DMC.

References

[1] Mahmoodabadi, R. G. *et al.* Point spread function in interferometric scattering microscopy (iSCAT). part i: aberrations in defocusing and axial localization. *Optics Express* **28**, 25969 (2020).

[2] Kashkanova, A. D., Blessing, M., Gemeinhardt, A., Soulat, D. & Sandoghdar, V. Precision size and refractive index analysis of weakly scattering nanoparticles in polydispersions. *Nature Methods* **19**, 586–593 (2022).

[3] Busatto, S. *et al.* Brain metastases-derived extracellular vesicles induce binding and aggregation of low-density lipoprotein. *Journal of Nanobiotechnology* **18** (2020).

[4] Busatto, S. *et al.* Considerations for extracellular vesicle and lipoprotein interactions in cell culture assays. *Journal of Extracellular Vesicles* **11** (2022).
