# Supplementary figures and images for: Label‐free discrimination of extracellular vesicles from large lipoproteins
Source: J Extracell Vesicles. 2023 Jul 25;12(8):12348. doi: 10.1002/jev2.12348 (PMC10366660; doi:10.1002/jev2.12348)

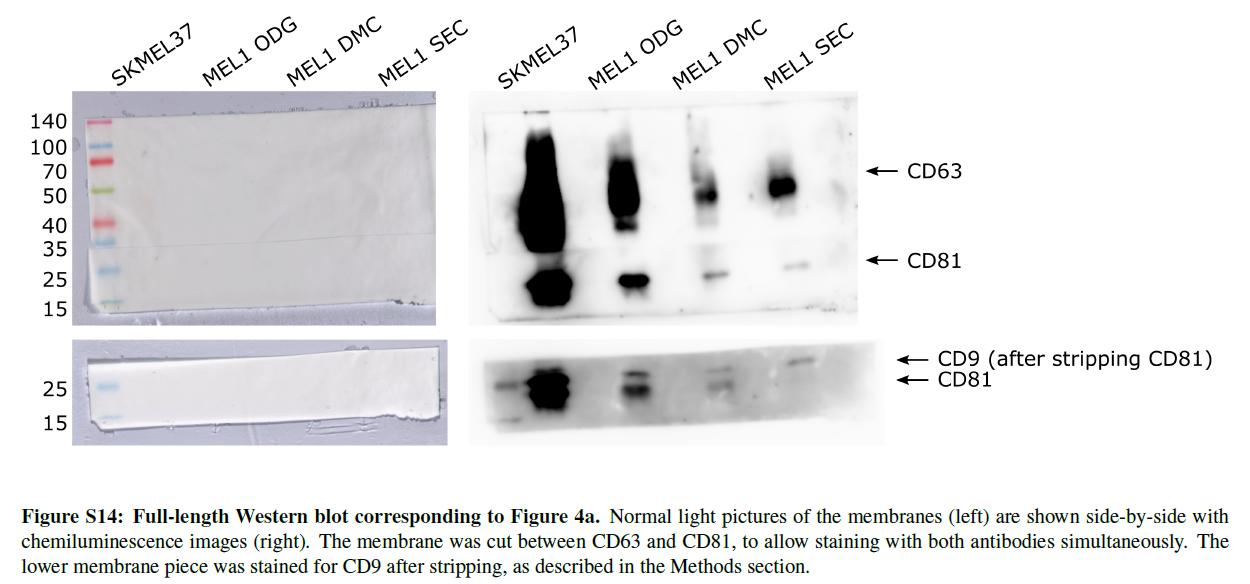

Supplement: Supplementary file 2 — Supporting Information [file JEV2-12-12348-s002.png]
